# Cortical Neural Plastic Changes Post-stroke Using Bicephalic Transcranial Direct Current Stimulation: A Prospective Non-randomized Study

**DOI:** 10.7759/cureus.88586

**Published:** 2025-07-23

**Authors:** Ashu Bhasin, Gulafshan Iqbal, Rahul Sharma, Senthil S Kumaran, Vishnu VY, Padma V Srivastava

**Affiliations:** 1 Neurology, All India Institute of Medical Sciences, New Delhi, IND; 2 Nuclear Magnetic Resonance (NMR), All India Institute of Medical Sciences, New Delhi, IND

**Keywords:** functional mri (fmri), neuronal plasticity and repair, physiotherapy management, post-stroke recovery, transcranial direct current stimulation (tdcs)

## Abstract

Background

Transcranial direct current stimulation (tDCS) has attracted attention among researchers as it has significant neurorehabilitative effects post-stroke. The purpose of the study was to investigate the safety, feasibility, and probable efficacy of bicephalic tDCS in improving hand function after stroke.

Methods

We conducted a prospective non-randomized controlled study involving two groups of participants with chronic stroke. Participants were allocated to real tDCS (r-tDCS) and age-matched controls to sham tDCS (s-tDCS). The treatment session was for 20 minutes, along with physiotherapy for four weeks. The primary outcome measure was the Fugl-Meyer assessment (FMA), while secondary outcomes were the Action Research Arm Test (ARAT), modified Barthel Index (mBI), Brunnstrom stage, and functional magnetic resonance imaging (fMRI) measures. All the assessments were done at baseline, four weeks, and three months.

Results

Ninety-five patients were recruited. No side effects were reported. FMA (d=0.75; 95% CI: 1.17 to 0.34; p=0.03) and mBI (d=0.70; 95% CI: 1.11 to 0.19; p=0.04) showed significant improvement at three months, although mean FMA was higher in r-tDCS than s-tDCS at four weeks. ARAT and Medical Research Council (MRC) were statistically insignificant between the groups at all time points. A mild increase in the cluster counts of premotor and primary motor cortex (Brodmann area (BA) 4 and BA 6) in group 1 was observed at four weeks and three months.

Conclusion

The novel intervention tDCS was safe and compliant among subjects but failed to report a significant efficacy between the two groups. A greater neural activation of ipsilesional primary motor regions (BA 4 and BA 6) and a weak positive correlation of FMA with laterality index (LI) were observed in the r-tDCS group than the s-tDCS group which may have led to improved hand function.

## Introduction

Non-communicable diseases (NCDs), like stroke, remain a significant public health challenge, imposing significant morbidity and increased dependence on family and caregivers [1]. Due to rapid urbanization, demographic changes, socioeconomic advancements, and environmental factors, global disease burden suggests that deaths from stroke will increase by 50% with the bulk of the burden residing in low- to middle-income countries [2]. 

The insult primarily leads to motor, sensory, balance, and gait deficits with more than 80% of survivors experiencing impaired hand function [3]. Neurorehabilitation is an imperative goal of treatment for stroke-related motor deficits. Task-oriented training based on motor learning principles, noninvasive cortical stimulation, and assistive technologies to restore functional movements has proven to be beneficial in post-stroke impairments [4].

Transcranial direct current stimulation (tDCS) is a promising therapeutic modality that enables the alteration of cortical excitability by passing direct currents causing hypo- or hyperpolarization of neuronal resting membrane potentials. Recent studies have administered high-definition tDCS (HD-tDCS) with single- or multi-channel montage application. The bicephalic tDCS with anode applied on the affected cortex, especially M1, and cathode over the non-affected cortex is an efficient technique to minimize the transcallosal inhibitory influx and normalize electrical impulses from corticospinal tracts (CST), and this technique of electrode placement is superior to other montages in recent trials [5].

Functional magnetic resonance imaging (fMRI) is specific in elucidating neural correlation with specific motor tasks for brain functions and is a surrogate marker for diagnosis and therapeutic decision-making [6]. Few studies have commented on the patterns of cortical excitability of primary motor cortex (M1) with anodal tDCS proving its worth to use the montage for better functional gains [7]. The objectives of this research were primarily to investigate the safety, feasibility, and efficacy of tDCS in improving hand function in chronic stroke and secondarily to explore its effects on blood oxygenation-level-dependent (BOLD) imaging through fMRI at four weeks and three months.

## Materials and methods

Sample size

The sample size was calculated using G*Power (Heinrich-Heine-Universität Düsseldorf, Düsseldorf, Germany) with a power estimate of 80% (significance level of 0.05). With an effect size of 0.6, referring to the previous trials [8], we calculated the sample size using (α, β) type I and type II errors: $\text{n}=\left[ \frac{\left( \frac{\text{Z1-&alpha;}}{2} \right)+\text{Z1-&beta;}}{\text{&delta;}} \right]^{2}&times;\text{2\sigma}^{2}$. Here, δ is the previous mean difference, and σ is the standard deviation, which revealed a minimum of 37 subjects in each group. Estimating the dropout rates and discontinuation of treatment, we recruited patients more than the minimum accepted number in each group.

Inclusion and exclusion criteria

The inclusion and exclusion criteria are listed in Table 1. Few contraindications to tDCS and fMRI like cranioplasty, diagnosed epilepsy, implantable medical devices like pacemakers and defibrillators, surgical metal objects like clips, and dermatological illness were also excluded.

**Table 1:** Inclusion and exclusion criteria of the study fMRI: functional magnetic resonance imaging; tDCS: transcranial direct current stimulation

Inclusion criteria
(i) Diagnosed stroke with index event from 3 months to 2 years
(ii) Age 18-75 years
(iii) Both ischemic and hemorrhagic stroke
(iv) Power of hand muscles (1-3)
(v) Brunnstrom stage (2-4)
(vi) Conscious and comprehensible
Exclusion criteria
(i) Progressive neurological deficits
(ii) Cognitive worsening
(iii) Malignancies
(iv) Diagnosed epilepsy
(v) Implantable medical devices like pacemakers and defibrillators
(vi) Surgical metal objects like clips and dermatological illness that prohibited the fixation of electrodes
(vii) Ongoing pregnancy and lactation
(viii) Few contraindications to tDCS and fMRI such as cranioplasty

Design

We conducted a non-randomized, double-blinded (patient and assessor) study at All India Institute of Medical Sciences, New Delhi, India. One of the assessors was blinded to the treatment outcomes.

Groups

Participants were divided into two groups: intervention or real tDCS (r-tDCS) and conventional physiotherapy as group 1 and control/placebo or sham tDCS (s-tDCS) and conventional physiotherapy as group 2.

Allocation

Participants followed a 1:1 sequential allocation as they arrived in the outpatient department (OPD) to groups 1 and 2.

Protocol

**Figure 1:**
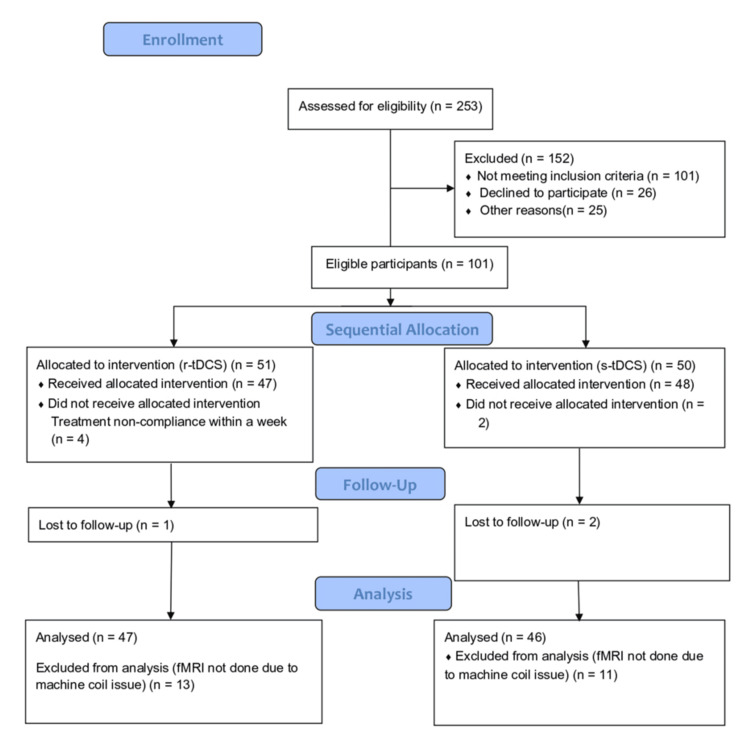
TREND flowchart of the study TREND: Transparent Reporting of Evaluations with Nonrandomized Designs; r-tDCS: real transcranial direct current stimulation; s-tDCS: sham transcranial direct current stimulation; fMRI: functional magnetic resonance imaging

The protocol was ethically approved by the Institute Ethics Committee (IEC) of All India Institute of Medical Sciences (AIIMS) (approval number: IEC-229/11.04.2020). All patients were screened from the neurology OPD clinics of the institute, and verbal and written informed consent was taken prior to recruitment from all eligible patients. The details of the study design are reported in accordance with the Transparent Reporting of Evaluations with Nonrandomized Designs (TREND) guidelines (Figure 1). The primary outcome measures were change in the Fugl-Meyer assessment (FMA) for the upper limb at four weeks and three months, while the secondary outcomes were change in the Action Research Arm Test (ARAT), modified Barthel Index (mBI), Brunnstrom stage of stroke recovery, and fMRI measures like BOLD mapping and cluster counts in the lesioned motor areas along with the laterality index (LI) of motor cortex at same time points. 

For r-tDCS (Mind Acquity, Walnut Medical Pvt. Ltd., Mohali, Punjab, India), the following specifications were used: current range 0.01-2 mA, voltage 2 mV, digital-to-analog converter (DAC) resolution 12 bit, and correction time 10 ms. The surface electrodes were soaked in normal saline with the anode placed over the lesioned motor cortex C3 for the left brain to stimulate the primary motor area (Brodmann area (BA) 4 and BA 6). The cathode was placed over the contralateral or non-lesioned cortex C4 for the right brain. The 2 mA current ran through the brain, completing the circuit for 20 minutes for three weeks (five days/week), accounting for 15 sessions. The online mode of tDCS with exercise regimen was adopted, where the stimulation and physiotherapy regimen were practiced simultaneously [9]. Few tailored exercises were fist making, pinch and spherical grasp, wrist extension, and functional activities like drinking a glass of water, opening a water can, and folding a paper. The electrode placement for s-tDCS remained the same, with the current initially started for two minutes, followed by a ramp down, and then switched off for an entire 18 minutes with the electrodes fixed at required points.

Physiotherapy protocol

A physiotherapist administered physiotherapy to participants and was aware of the stimulation conditions. The goals of the regimen were to facilitate and strengthen the weak muscles, to inhibit the tone and hyperreflexia, to attain controlled, rhythmic, and purposeful movement, to retrain functional goals and set achievable targets, to augment vocational training, and to be independent. The training session lasted 45 minutes, five days a week, for four weeks [10]. Mandatory 20 sessions were administered to all patients. If the patient does not come for treatment for two consecutive weeks, he will be treated as a dropout. Therapy was adjusted to the clinical assessment of each patient. Some of the common exercises were as follows: cryotherapy followed by active stretching for a minimum of 10 seconds to a maximum of 30 seconds for wrist long flexors, elbow flexors, shoulder adductors and flexors, and small hand muscles; motor learning techniques; facilitation of hand muscles; stroking, brushing, weight-bearing, and passive movements of affected muscles (3-5 strokes to facilitate); task-oriented training (TOT); picking up a tool and a glass of water, pen holding, and bottle opening; lower extremity exercises consisting of stretching, range of motion, strengthening, and weight-bearing activities; and balance and gait re-education at home. 

fMRI

BOLD data were acquired using the echo planar imaging (EPI) sequence using a 3T MR scanner (Achieva, Siemens Medical Solutions, Erlangen, Germany) with a standard head coil. Block design with alternate baseline and activation cycles was used with a total of 90 whole-brain EPI measurements (timed repetition (TR)=1000 ms; timed echo (TE)=25 ms; slices=45; slice thickness=4 mm). Preprocessing included motion correction, spatial normalization using cost-function masking to account for lesions, and spatial smoothing with a 6 mm Gaussian kernel. Lesion masks were traced on T1-weighted images and used during normalization to minimize warping artifacts. Motion parameters, white matter, and cerebrospinal fluid (CSF) signals were regressed out to minimize noise.

Motor paradigm

The subjects were asked to perform the motor task with the paretic/affected hand, with self-paced (minimum 0.5 Hz) fist clenching/extension of the wrist joint depending upon the extent of motor damage. For healthy controls, the dominant and non-dominant hand motion was used.

Statistical analysis

All data were analyzed using IBM SPSS Statistics for Windows, Version 25.0 (IBM Corp., Armonk, New York, United States). Baseline characteristics of participants were compared between groups using the independent t-test. Paired t-tests were used to compare means and medians within the groups. Multiple measures analysis of variance (ANOVA) and two-sample t-tests were used between groups when two or more different means were compared at different time points. The statistical significance level was p=0.05. Postprocessing of fMRI data was analyzed using the SPM2 software (Wellcome Department of Cognitive Neurology, London, United Kingdom) running under the MATLAB environment (MathWorks, Natick, Massachusetts, United States). The functional images were realigned, normalized, and then smoothed by a 6 mm Gaussian filter before statistical analysis. Montreal Neurological Institute (MNI) coordinates were co-related with Talairach's Atlas for the gray and white matter areas of the brain. The volume of the lesion was analyzed by ImageJ (Version 1.42q, Wayne Rasband, National Institutes of Health, Bethesda, Maryland, United States).

## Results

**Figure 2:**
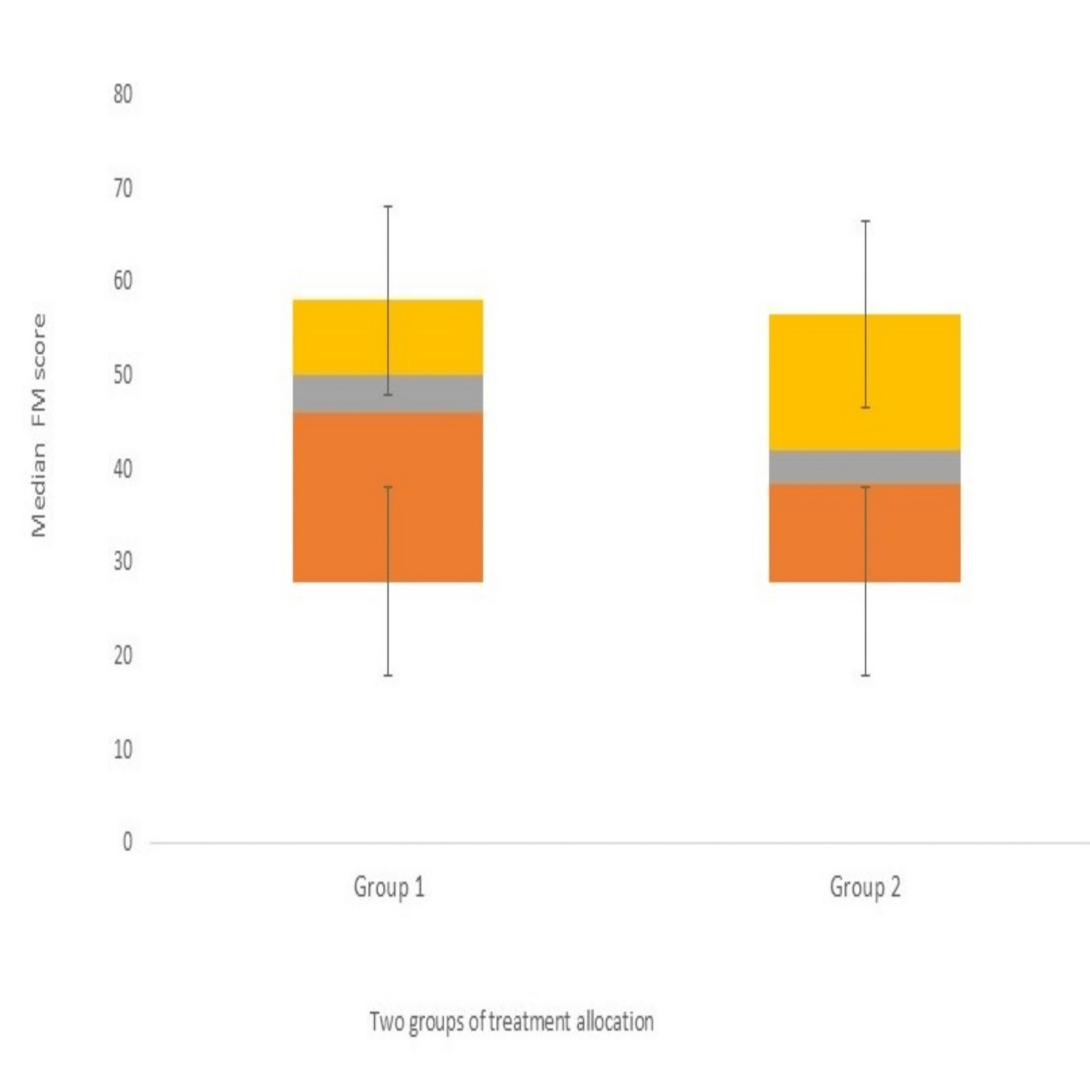
Median FM scores at three months in groups 1 and 2 FM: Fugl-Meyer

The baseline characteristics were comparable in age, sex, duration after stroke, presence and severity of disease, or baseline motor scores (at p>0.05) (Tables 2-3). The total number of patients screened from the neurology OPD and wards was 253, and 95 were recruited. The median age of group 1 was 53+13.2 years, and that of group 2 was 48+10.2 years. The mean time of onset was 20.5 months, with 58 ischemic and 37 hemorrhagic strokes. Median values of FMA and ARAT were high in group 1 as compared to group 2 at four weeks, indicating a trend of improvement with real cortical stimulation in impairment scales (Figure 2). Two-sample t-tests revealed no significant differences between groups 1 and 2 in FMA (95% CI: 0.89 to -0.07; Cohen's d=0.8), mBI (95% CI: 0.21 to -0.59; d=-0.19), and ARAT (95% CI: 0.47 to -0.33; d=0.06) at four weeks though FMA (d=0.75; 95% CI: 1.17 to 0.34; p=0.03) and mBI (d=0.70; 95% CI: 1.11 to 0.19; p=0.04) showed significant improvement at three months indicating a statistically and clinically meaningful difference in favor of group 1. MRC, ARAT, and Ashworth tone grade were statistically insignificant between the groups at all time points (p>0.05) (Table 4).

**Table 2:** Demographic details and risk factors of both groups with p-values CAD: coronary artery disease

	Group 1	Group 2	P-value
No. of subjects (N)	47	48	-
Male	31	29	0.89
Female	16	19	0.45
Median age	53+13.2	48+10.2	0.54
Risk factors			
Hypertension	32	33	0.45
Diabetes mellitus	28	28	0.34
Cholesterolemia	23	21	0.26
Smoking	22	20	0.89
Alcohol consumption	25	18	0.99
Tobacco abuse	12	9	0.47
CAD	19	21	0.62
Type of stroke			
Ischemia	30	28	0.49
Hemorrhage	17	20	0.43
Time of onset (mean months)	21.3	19.4	0.43
Toast classification			
Large artery	12	9	-
Small vessel	11	13	-
Cardioembolic	9	10	-
Other determined strokes	7	8	-
Undetermined etiology	8	8	-

**Table 3:** Baseline clinical scores in stroke subjects segregated to groups 1 and 2 MRC: Medical Research Council grade for power; FMA: Fugl-Meyer assessment; mBI: modified Barthel Index; NIHSS: National Institutes of Health Stroke Scale; mRS: modified Rankin score; ARAT: Action Research Arm Test

Clinical scores	Group 1	Group 2	P-value
MRC (/5)	2.1+0.03	2.5+1.3	0.54
FMA (/66)	29.1+8.9	26.1+6.9	0.32
mBI (/100)	47.3+12.5	51.6+11.6	0.66
Brunnstrom stage of hand recovery (/6)	2.6+0.6	3+0.8	0.43
Median NIHSS at stroke onset	17+4.1	14+3.3	0.56
mRS at 3 months of the index event	3+0.9	3+0.5	0.32
ARAT (/57)	41.3+9.9	43.3+10.5	0.39

**Table 4:** Clinical outcome measures at four weeks and three months in groups 1 and 2 MRC: Medical Research Council grade for power; FMA: Fugl-Meyer assessment; mBI: modified Barthel Index; NIHSS: National Institutes of Health Stroke Scale; mRS: modified Rankin score; ARAT: Action Research Arm Test

	Group 1	Group 2	P-value	Group 1	Group 2	P-value
	4 weeks			3 months		
MRC	3.1+0.03	2.9+0.03	0.65	3.6+1.04	3.8+1.03	0.77
FMA	47.8+8.2	43.6+9.1	0.32	59.9+10.1	52.6+9.3	0.03
mBI (/100)	69.4+8.3	71.2+10.2	0.69	79.3+9.1	72.9+9.2	0.04
Brunnstrom (/6)	3.1+0.02	3.6+1.1	0.34	6.2+1.1	4.21+1.3	0.06
ARAT (/57)	44.8+9.5	44.2+7.9	0.53	47.5+7.2	49.1+9.1	0.66

Safety and feasibility

No serious/adverse events were reported. A few patients complained of scalp irritation after the stimulation. The current stimulation was well tolerated by patients.

fMRI results 

**Figure 3:**
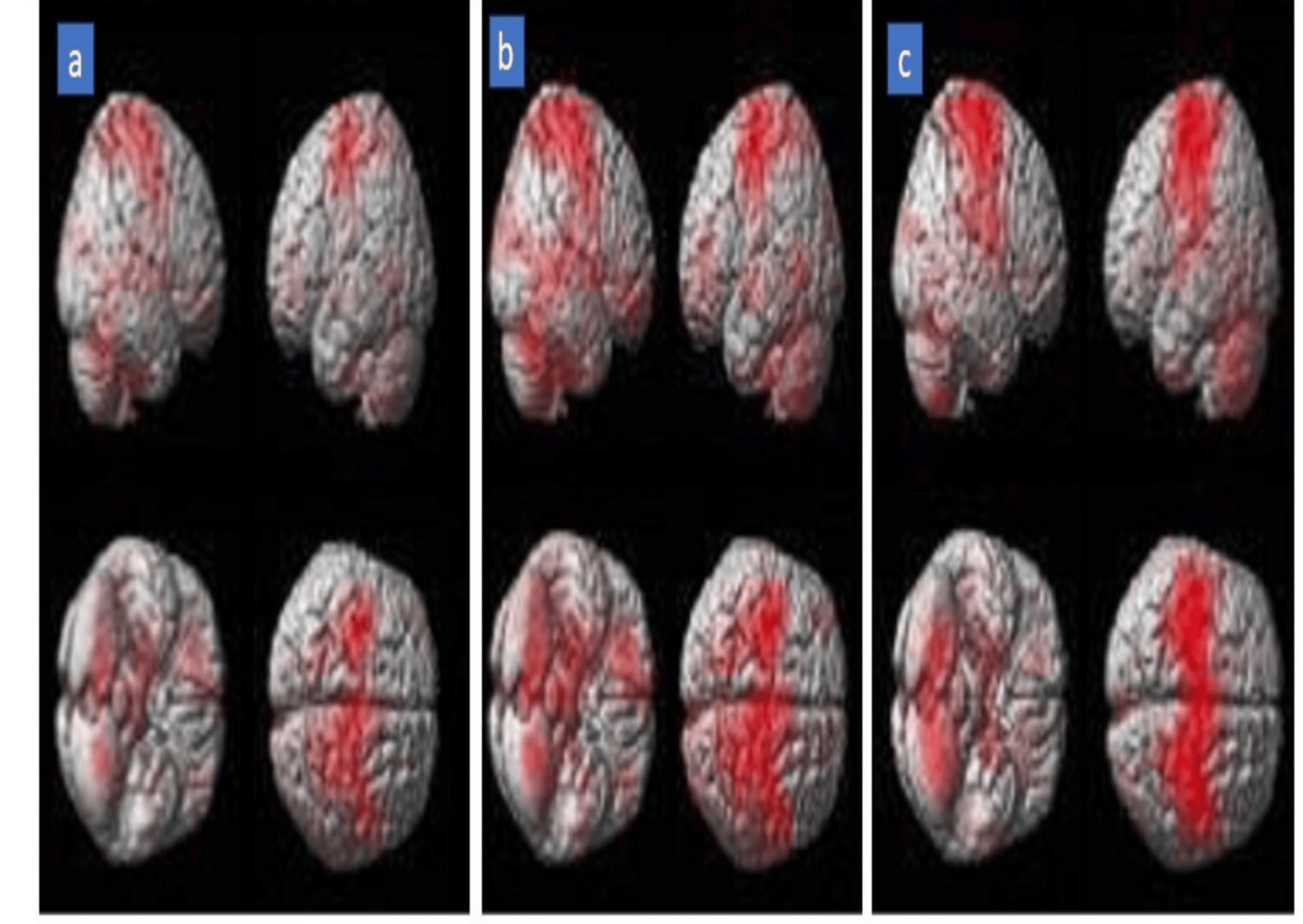
Three-dimensional rendered BOLD brain images showing (a) baseline and post-treatment cortical activation at (b) four weeks and (c) three months BOLD: blood oxygenation-level-dependent

The fMRI analysis of 40 subjects (120 datasets) was done through group analysis (contrast post-pre, significance level at 0.05). There was an increase in the cluster counts of dorsal premotor and primary motor cortex at four weeks and three months in r-tDCS versus s-tDCS. Right BA 6 (342 clusters), sensory cortex BA 2 and BA 3 (356 clusters), and inferior parietal lobule BA 40 (267 clusters) were active in group 1 as compared to group 2 (post-pre) (Table 5 and Figure 3).** **

**Table 5:** Group analysis of fMRI between groups 1 and 2 BA: Brodmann area; Rt: right; Lt: left; inf: inferior; fMRI: functional magnetic resonance imaging

No. of voxels	Z score	Area of activation	BA
342	4.04	Rt precentral gyrus	BA 6
193	3.78	Lt cingulate	BA 31
128	3.15	Lt insula, hippocampus	BA 25
356	2.90	Rt post central gyrus	BA 3 and BA 2
267	2.77	Rt inf parietal lobule	BA 40

**Figure 4:**
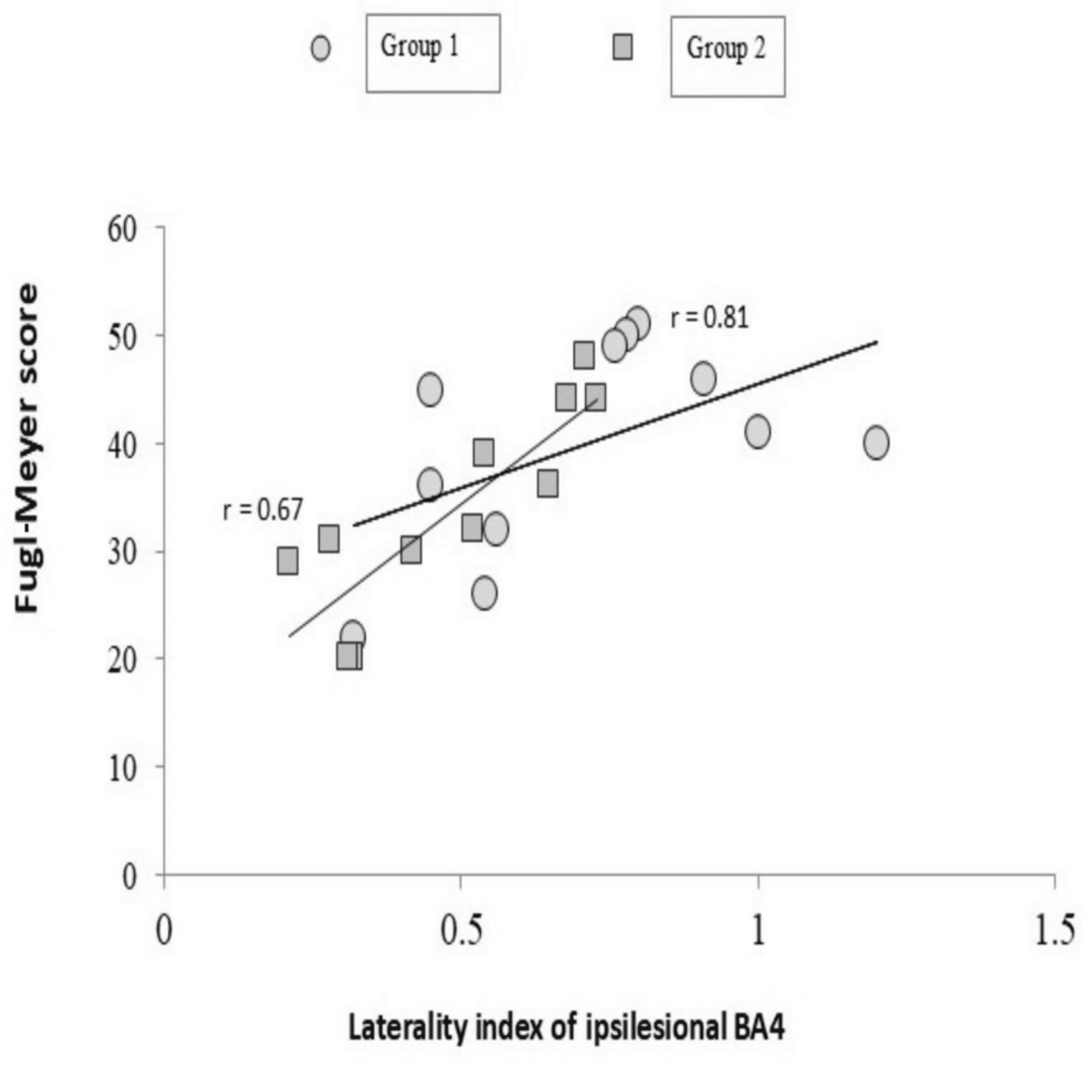
Correlation of LI and FM of both groups at four weeks LI: laterality index; FM: Fugl-Meyer; r: Pearson's coefficient; BA: Brodmann area

LI correlation with FM score showed linear positive correlation in group 1 with Pearson's correlation coefficient (r=0.81) than group 2 (r=0.67) at four weeks after the stimulation (Figure 4). Bilateral hand activation in both groups at four weeks showed that cerebellar, premotor cortex, and cognitive execution areas (BA 39, BA 40, and BA 10) were actively increased post-stimulation with tDCS and physiotherapy. The hemodynamic response curves for active and rest cycles were generated for each patient, and it was observed that the signal intensity of the lesioned cortex improved in r-tDCS as compared to sham at four weeks (95% CI: ~2.4 to 1.3; p=0.002). 

## Discussion

Our findings suggest that direct current stimulation in stroke patients is both safe and feasible, as none of the participants experienced any adverse events (such as itching, rash, or burns) or serious adverse events (such as seizures). The findings of the present study do not directly indicate the significant effects; nevertheless, it does not preclude us from stating that bihemispheric tDCS in chronic patients is a novel therapeutic vehicle to promote recovery post-stroke. Most recent randomized trials have shown a Fugl-Meyer assessment of the upper extremity (FMA-UE) improvement rate with real stimulation compared to sham stimulation (20.7-35.2% vs. 3.2-6.6%) [11]. We also observed significant improvement in impairment and disability scales (FMA, mBI, and Brunnstrom stage) in group 1 as compared to group 2 which lasted six months. The neurophysiology of transcallosal inhibition from the unaffected cortex provides a framework for tDCS application by upregulating the excitability of the ipsilesional motor cortex and/ or downregulating the contralesional motor cortex excitation [12]. Proof-of-concept studies have shown both approaches to have beneficial effects on motor skills and motor learning. This novel bicephalic stimulation design used in our study allowed us to test the effectiveness of simultaneously stimulating both the motor hemispheres to dysregulate the inter-hemispheric imbalances after stroke [13]. The rate of improvement in FMA score was greater from baseline to four weeks (stimulation plus physiotherapy) than the placebo group (48.4% vs. 35.5%) at four weeks, emphasizing that neuromodulatory cortical networks may be enhanced with r-tDCS (Figure 2) [14,15].

fMRI has been extensively used for studying the neural correlates of brain functioning through changes in blood oxygenation and flow [16]. Our previous studies have proven fMRI as a surrogate marker reflecting hand motor function with large datasets [17,18]. In the present study, with the limited number of BOLD scans, analysis may not be a direct representation of the outcomes, although it could show an increased cluster count in ipsilesional right BA 6 and BA 4 in group 1 as compared to group 2 at four weeks (group analysis post-pre, threshold 0.05). Incidentally, we also observed the inferior parietal lobule being active with 156 voxel counts, forcing us to perceive tDCS as a biofeedback device on the skull, activating the spatiocortical inputs targeting the affected pre-/perimotor area, enhancing the task performance [19,20]. The tDCS stimulates the sensory nerve endings under the electrodes, thereby modulating the resting membrane potential depending on excitation and inhibition protocols [21,22]. This sham neurophysiological principle gives insight into the pronounced activation of functional sensory cortical areas (BA 2 and 3) also after anodal stimulation.

We tried to establish the neural gains from stimulation and changes in the motor task. Normally, LI ranges from +1 (ipsilesional) and -1 (contralesional). A weak positive correlation of FMA and ipsilesional LI was observed in group 1 (95% CI: 2.4 to 0.9; r=0.81) than group 2 (95% CI: 1.2 to -0.7; r=0.79) (Figure 4). Restorative interventions like cognitive motor training, robotics and brain-computer interface (BCI) systems, stem cells, and growth factors have been widely explored in meta-analyses and shown remarkable treatment effect sizes like 0.34 (constraint-induced movement therapy (CIMT)), 0.55 (repetitive transcranial magnetic stimulation (TMS)), 0.65 (robotic arm training), and 0.92 (selective serotonin reuptake inhibitors) [23,24]. The mathematical transfer function model of the neurovascular coupling between local neural activity and the corresponding BOLD fMRI signal is called the hemodynamic response function (HRF) [25]. With an increased cluster count observed in group 1, it can be hypothesized that real stimulation may have led to an increased performance and a higher signal intensity reported from the HRF curves. Recovery after stroke, measured with fMRI connectivity models, shows that modulation of long-term connections between different regions within and outside the lesional area is associated with factors like demographics, lesion size, topography, and structural brain formations, which are tailored to individuals [26].

The combination of anodal excitatory and cathodal inhibitory tDCS and neuromotor training enhances motor activation and skill acquisition, leading to changes in the intrinsic and extrinsic milieu of a stroke-injured brain. Previous studies inferred that the combination of bihemispheric tDCS with motor learning improved or partly restored functional connectivity (fc) and resting-state fMRI (rs-connectivity) with lasting tDCS effects [27]. 

Group 2, who received sham stimulation along with a physiotherapy regimen, also showed some improvement. All patients had undergone repetitive task-oriented training for four weeks, stating that practice of an activity strengthens the brain networks at microscopic levels, which is evident through clinical scores and fMRI [28]. Task-based training produced statistically significant as well as clinically meaningful enhancement in the dexterous hand movements of subacute stroke survivors than conventional therapy. In the control group, some fMRI changes in the premotor cortex and in the bilateral posterior cerebellar hemispheres were observed than at baseline, suggesting that recruitment of sensorimotor cortices and the cerebellum contributes to recovery after exercise therapy.

ARAT is a score of 19 subsets of arm motor function with a total score of 57. Each test is given an ordinal score of 0, 1, 2, or 3, with higher values indicating better arm motor status. The FMA-UE is a 66-ordinal point scale that assesses the impairment level of a stroke-injured subject with the subscales measured on flexion and extensor synergies, wrist and hand function, reflexes, and coordination [29]. We observed an improvement in FMA-UE at three months but not in ARAT as the latter has a ceiling effect, i.e., patients with mild impairment might already score near the top, limiting the test's ability to detect group differences. Patients may show significant gains in motor control (FMA) but not yet regain functional task ability (ARAT). Functional gains in ARAT tend to lag behind motor impairment recovery, i.e., FMA.

With the high spatial resolution that fMRI offers across the brain, research has shown that tDCS effects not only are spatially restricted to the region underneath the stimulating electrode but also modulate the entire brain network. The combination of fMRI and tDCS provides unique insight into the neuromodulatory effects of tDCS not only in the targeted brain regions but also in their interconnected networks. The ultimate aim of these mechanistic experiments is to find a causal relationship between the clinico-behavioral and neural responses to tDCS. In the present paper, we tried to bring to the readers the effects of tDCS in chronic stages of stroke [30] and reported its safety, durability, and compliance among stroke patients but deter to comment on the significant efficacy of tDCS in stroke. 

Limitations

As no research can be without flaws, we also admit to having some of them. Being a non-randomized study, an unblinded physiotherapist who knew the treatment groups, and lack of complete fMRI datasets do not predict a casual relationship between the intervention and outcomes potentially leading to bias.** **The tDCS machine used in the present study was not a high-definition equipment, which might have tempered the results, and we hope all the limitations are overcome in the main primary trial by our group.

Future directions

A phase 2, three-arm randomized controlled trial (RCT) (CTRI/2024/06/068278) with the newly designed piezoelectric glove and high-definition (HD) tDCS will be commencing soon from our group. tDCS has opened wider rehabilitational modes of treatment, and several robust RCTs are underway using different current intensities, montages (unimodal vs. multimodal), high-definition equipment, coupling with electroencephalography (EEG)/near-infrared spectroscopy (NIRS)/fMRI, and biochemical correlates or markers to validate the efficacy of noninvasive brain stimulation in stroke. The combination of low costs, minimal safety risks, and the potential for tele-tDCS promotes its seamless integration into clinical practice.

## Conclusions

tDCS seems a safe and tolerable portable equipment for neurorehabilitation interventions. A structured physiotherapy regimen with or without tDCS improves impairment-related deficits. Neural plasticity induced by bicephalic tDCS exhibits minor changes at ipsilesional premotor and primary motor regions (BA 4 and BA 6) in real versus sham stimulation. However, no conclusive evidence of efficacy was established in the present study.
